# Laparoscopic gastrectomy with D2 lymphadenectomy for gastric cancer: initial Egyptian experience at the National Cancer Institute

**DOI:** 10.1186/s43046-020-00023-7

**Published:** 2020-02-18

**Authors:** Mohamed Aly Abdelhamed, Ahmed Abdellatif, Ahmed Touny, Ahmed Mostafa Mahmoud, Ihab Saad Ahmed, Sherif Maamoun, Mohamed Shalaby

**Affiliations:** Department of Surgical Oncology, National Cancer Institute, Cairo University, Cairo, Egypt

**Keywords:** Gastric cancer, Laparoscopic gastrectomy, D2 lymphadenectomy

## Abstract

**Background:**

Laparoscopic gastrectomy has been used as a superior alternative to open gastrectomy for the treatment of early gastric cancer. However, the application of laparoscopic D2 lymphadenectomy remains controversial. This study aimed to evaluate the feasibility and outcomes of laparoscopic gastrectomy with D2 lymphadenectomy for gastric cancer.

**Results:**

Between May 2016 and May 2018, twenty-five consecutive patients with gastric cancer underwent laparoscopic D2 gastrectomy: eighteen patients (72%) underwent distal gastrectomy, four patients (16%) underwent total gastrectomy, and three patients (12%) underwent proximal gastrectomy. The median number of lymph nodes retrieved was 18 (5–35). A positive proximal margin was detected in 2 patients (8%). The median operative time and amount of blood loss were 240 min (200–330) and 250 ml (200–450), respectively. Conversion to an open procedure was performed in seven patients (28%). The median hospital stay period was 8 days (6–30), and the median time to start oral fluids was 4 days (3–30). Postoperative complications were detected in 4 patients (16%). There were two cases of mortality (8%) in the postoperative period, and two patients required reoperation (8%).

**Conclusions:**

Laparoscopic gastrectomy with D2 lymphadenectomy can be carried out safely and in accordance with oncologic principles.

## Background

Gastrectomy with proper lymphadenectomy is considered the cornerstone of treatment for potentially curable gastric cancer [1]. Since the first laparoscopic-assisted gastrectomy was reported by Kitano et al. [2] in 1994, many studies have demonstrated that laparoscopic gastrectomy has advantages over open surgery, such as good cosmesis, reduced pain, and shorter hospital stays [3–5]. With the improvement in laparoscopic technology, laparoscopic gastrectomy has gained great popularity and has become the standard therapy in Korea and Japan for early gastric cancer [6].

As experience in laparoscopic gastrectomy for early gastric cancer has increased, some surgeons have become concerned about the use of laparoscopic D2 lymphadenectomy in gastric cancer [7]. Additionally, D2 lymphadenectomy is a more challenging procedure than D1 lymphadenectomy [8].

The extent of lymph node dissection is described according to the Japanese gastric cancer treatment guidelines. The draining lymph nodes of the stomach are divided into stations. Stations 1–6 are the perigastric lymph nodes along the lesser and greater curvature of the stomach. Stations 7–12 correspond to the lymph nodes around the left gastric artery (station 7), common hepatic artery (station 8), celiac trunk (station 9), splenic artery (stations 10 and 11), and hepatoduodenal ligament (station 12). Stations 13–15 are the lymph nodes behind the pancreas and along the superior mesenteric and the middle colic vessels. Station 16 refers to the para-aortic lymph nodes [9, 10].

D1 lymphadenectomy is defined as the removal of lymph node stations 1 to 7 in cases of total gastrectomy, while in D1 distal gastrectomy, lymph node stations 1, 3, 4sb, 4d, 5, 6, and 7 are removed. D2 total gastrectomy includes the removal of lymph node stations 1 to 12a, while D2 distal gastrectomy includes D1 lymphadenectomy + the removal of the 8a, 9, 11p, and 12a lymph node groups. D1 lymph node dissection is indicated in clinical T1N0 tumors, while D2 lymphadenectomy is indicated for clinical N+ or clinical T2-T4 tumors [9].

Many studies have compared laparoscopy-assisted gastrectomy with conventional open surgery; however, only a few studies have reported the results of laparoscopic D2 gastrectomy. The most important trials are the KLASS-02 trial that was conducted in Korea [11], the CLASS-01 trial from China [12], and the JCOG0912 trial that was performed in Japan [3]. In the KLASS-02 trial, a total of 1050 patients with locally advanced gastric cancer were randomly assigned to undergo either laparoscopic distal gastrectomy or open distal gastrectomy. The CLASS-01 trial included 1056 patients with clinical stage T2-T4aN0-3 gastric cancer who were randomized to undergo either laparoscopic or open D2 gastrectomy. In the JCOG0912 trial, Katai and coworkers included 921 patients with clinical stage IA/IB gastric cancer scheduled for either laparoscopy-assisted distal gastrectomy or open distal gastrectomy, and D2 lymphadenectomy was performed for stage IB (27.7% of patients). These studies concluded that laparoscopy-assisted D2 gastrectomy is safe, feasible, and not inferior to open surgery in terms of short-term outcomes. Despite these promising results, many doubts have been raised about the quality and oncologic safety of laparoscopic D2 lymph dissection compared to those of open surgery.

Within this context, the present study aims to report our initial experience with laparoscopic D2 gastrectomy regarding the oncologic feasibility, technical safety, and short-term outcomes.

## Methods

### Study design

This prospective interventional pilot study comprised 25 patients with gastric cancer who underwent laparoscopic D2 gastrectomy between May 2016 and May 2018. All patients provided informed consent. The study was approved by our institutional ethical committee (Institutional Review Board, IRB # 00004025), National Cancer Institute, Cairo University. The inclusion criteria were as follows: pathologically proven adenocarcinoma of the stomach, clinical T1 and T2 tumors discovered during preoperative gastroscopy and abdominal computed tomography, patients with T3 or T4 tumors or those with nodal involvement who had shown a good response according to the Response Evaluation Criteria In Solid Tumors (RECIST) criteria 1.1 (> 30% decrease in tumor size) after neoadjuvant chemotherapy, adenocarcinoma of the cardia that regressed post-neoadjuvant chemotherapy, and patients with Eastern Cooperative Oncology Group (ECOG) performance status 0–2. Patients with metastatic disease, those who underwent previous upper abdominal surgeries and those with poor ECOG performance status (3, 4) were excluded. A preoperative work-up that included upper gastrointestinal endoscopy and computed tomography was routinely carried out. Endoscopic ultrasound (EUS) was not routinely performed. Pathological staging was determined according to the 8th edition of the American Joint Committee on Cancer (AJCC) TNM classification [13].

The primary outcome was to evaluate the feasibility of laparoscopic gastrectomy with D2 lymph node dissection. The secondary outcomes were surgical and oncologic outcomes. Surgical outcomes, including operative time, blood loss, length of hospital stay, time until oral fluid intake, morbidity, and mortality, were evaluated. The oncologic outcomes included the total number of dissected lymph nodes, histopathologic data, and proximal and distal resection margins.

Patient demographics and histopathologic and perioperative outcomes were recorded prospectively in our department database and were analyzed.

Statistical analysis was performed using IBM SPSS® Statistics version 23 (IBM® Corp., Armonk, NY, USA). Numerical data were expressed as the mean and standard deviation or median and range as appropriate. Qualitative data were expressed as the frequency and percentage.

### Surgical procedure

D2 lymphadenectomy was always performed according to the lymph node classification of the Japanese Gastric Cancer Association [9]. The operation time was measured from the first skin incision to the closure of all skin incisions. Conversion to an open procedure was defined as any abdominal incision for any reason before finishing laparoscopic D2 lymphadenectomy. According to the location of the tumor, the laparoscopic procedure was either distal gastrectomy, proximal gastrectomy, or total gastrectomy. Distal gastrectomy was performed for lesions in the pylorus, total gastrectomy was used for lesions in the body or proximal lesions for which the distal stomach could not be preserved, and proximal gastrectomy was performed for lesions of the upper third of the stomach or the cardia.

**Fig. 1:**
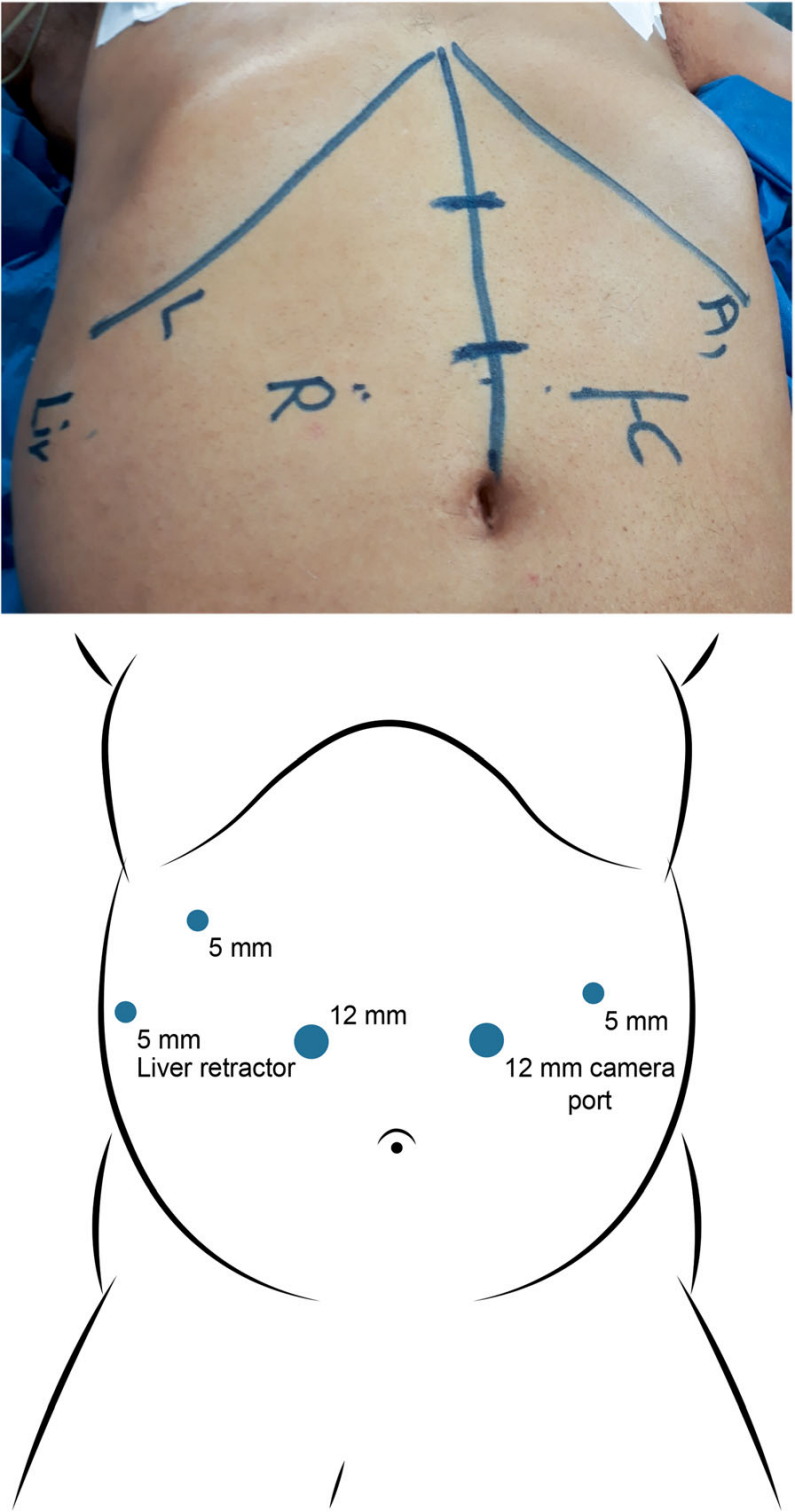
Port placement. C: camera port, R: right working port, L: left working port, A: assistant port, Liv: liver retractor port

The patient was placed in the supine position, and both arms were extended. General anesthesia was administered. An orogastric tube was placed to decompress the stomach, and a Foley catheter was inserted into the bladder. Intravenous antibiotics were injected within 30 min before the skin incision. Sequential stocking was applied to the lower extremities for deep vein thrombosis prophylaxis. The patient was secured to the surgical bed to facilitate a maximum reverse Trendelenburg position. The surgeon and person holding the camera stood on the patient’s right side. The first assistant and the scrub nurse were on the left side. The pneumoperitoneum was created with a closed technique using a Veress needle at the base of the umbilicus. The two working ports were placed in the right upper quadrant, and the other two ports were placed in the left upper quadrant for the person holding the camera and the assistant. The fifth port for liver retraction was inserted in the right anterior axillary line (Fig. 1). The abdomen was explored for evidence of liver or peritoneal metastatic disease, and peritoneal cytology was routinely performed. After the assistant retracted the liver upward using the right anterior axillary port, dissection started at the lesser curvature of the stomach, and the lesser omentum was divided with LigaSure™ (Covidien, Dublin, Ireland) as close to the liver as possible, thus reaching the cardia and the right crus of the diaphragm. The gastrocolic ligament was divided along the border of the transverse colon using LigaSure™.

**Fig. 2:**
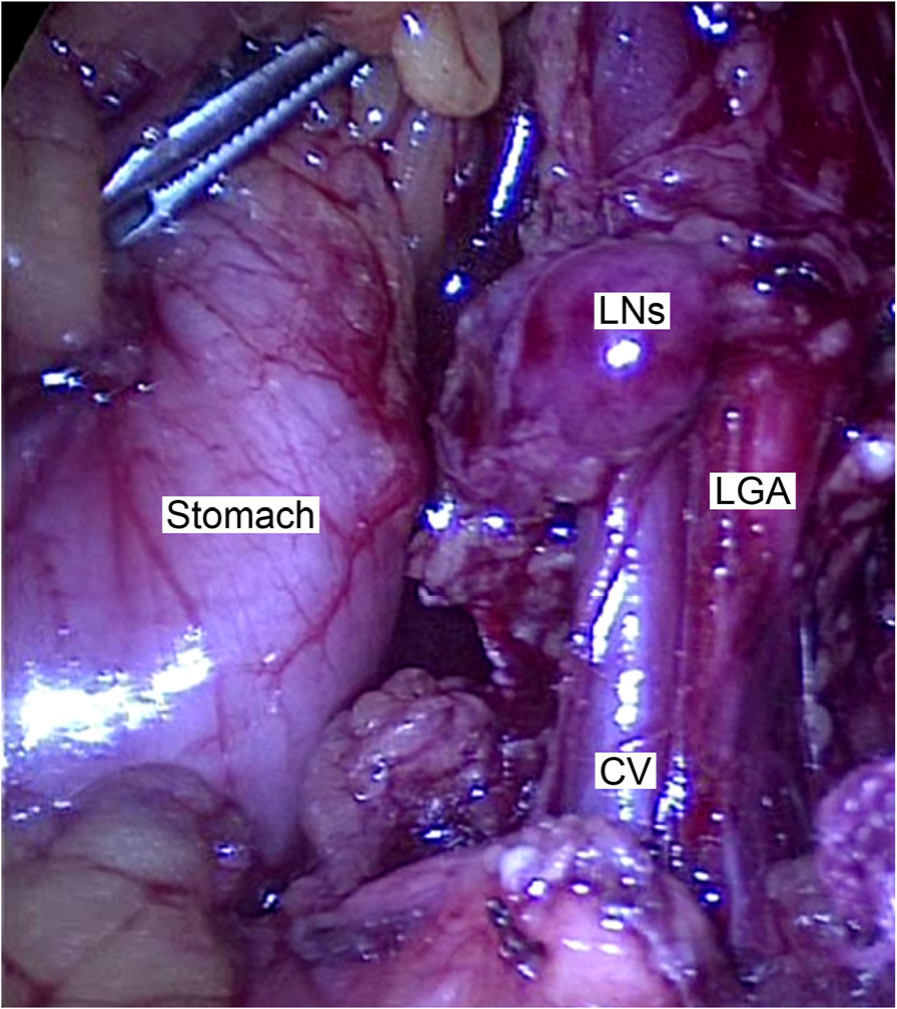
Lymph nodes along the left gastric artery (group 7). LGA: left gastric artery, CV: coronary vein, LN: lymph node

**Fig. 3:**
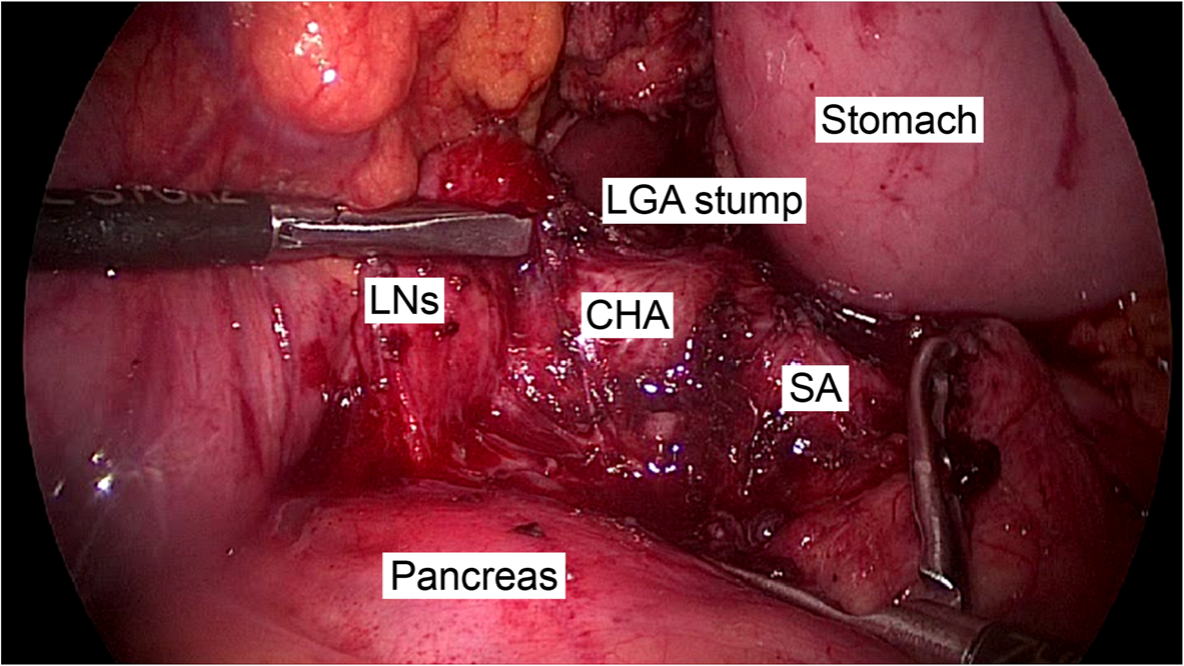
Dissection of lymph node groups 7, 8a, 9, 11p. CHA: common hepatic artery, SA: splenic artery, LGA: left gastric artery, LNs: lymph nodes

By flipping the stomach upward and retracting the pancreas downward to expose the upper border of the pancreas, lymph node dissection along the proximal splenic artery (group 11) was initiated after opening the anterior pancreatic capsule and entering the anterior pancreatic space, followed by the dissection of the left gastric artery (group 7) (Fig. 2) and celiac lymph nodes (group 9). The left gastric vein was controlled using 10 mm double endoclips, and the left gastric artery was also controlled using 10 mm double endoclips. Subsequently, dissection was continued in order to remove the nodes along the common hepatic artery (group 8a) (Fig. 3) and was continued until the hepatoduodenal ligament was reached (group 12a); then, the right gastric artery was controlled as it originated from the hepatic artery (group 5).

After complete separation of the greater omentum from the transverse colon, the right gastroepiploic vein was divided with LigaSure™, and the right gastroepiploic artery was exposed and divided at its origin from the gastroduodenal artery between the 10 mm endoclips just above the pancreatic head for the dissection of the infrapyloric lymph nodes (group 6).

Passing into the tunnel below the pylorus, the inferior wall of the first part of the duodenum was denuded near the surface of the pancreatic head to create a retroduodenal tunnel. Then, the duodenum was transected using a 60-mm linear stapler (blue stapler load). The dissection of the gastrocolic ligament was continued toward the spleen, and the left gastroepiploic artery was divided using LigaSure™. Before gastric transection, the right paracardial lymph nodes (group 1) and lymph nodes along the lesser curvature were dissected. The stomach was then transected using sequential firings of a 60-mm linear stapler after taking an appropriate margin proximal to the tumor. The 12 mm stapling port was enlarged, and the specimen was withdrawn through the wound. For the total gastrectomy procedure, all steps were performed the same as in the distal gastrectomy procedure, in addition to the mobilization of the distal esophagus and the removal of lymph node groups 2, 10, and 11d as well as all 4sa lymph nodes.

**Fig. 4:**
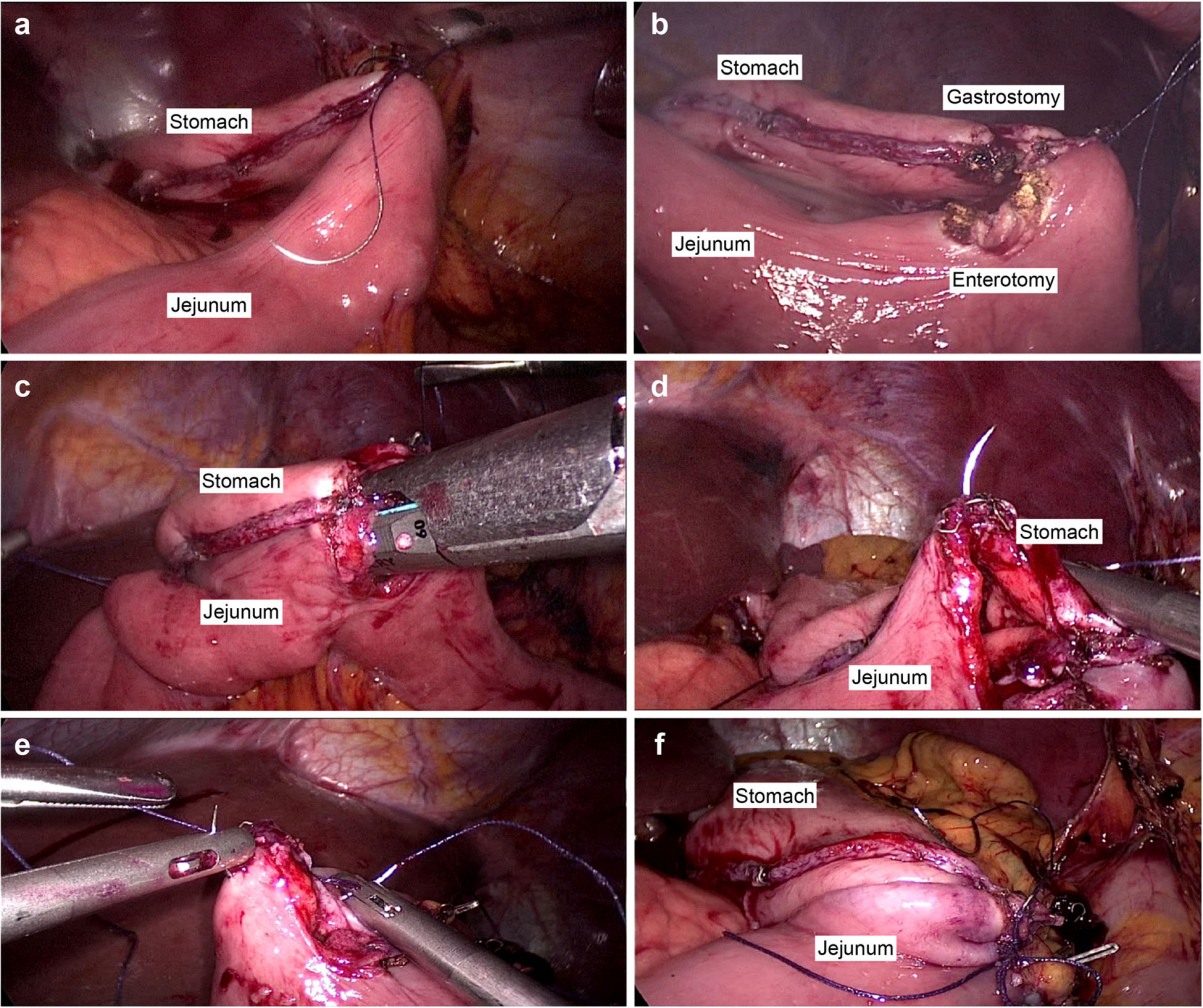
Billroth II gastrojejunal anastomosis. **a** The proximal jejunum was approximated to the proximal gastric pouch with a traction suture. **b** Enterotomies in the jejunum and stomach. **c** Stapler was inserted through enterotomies in the jejunum and stomach. **d**, **e**, **f** The stapler defect was closed in two layers

Reconstruction after distal gastrectomy was performed either with intracorporeal Billroth II anastomosis (total laparoscopic distal gastrectomy) (Fig. 4) or with extracorporeal Roux-en-Y anastomosis through a 5–7-cm transverse incision (laparoscopy-assisted distal gastrectomy), while reconstruction following total gastrectomy was performed extracorporeally with Roux-en-Y anastomosis through a small laparotomy incision below the xiphoid process (laparoscopy-assisted total gastrectomy).

For the tumors of the cardia for which the distal stomach could be preserved, proximal gastrectomy was performed. The gastrocolic ligament was divided with LigaSure™ while preserving the right gastroepiploic artery. Lymphadenectomy was carried out along the common hepatic, splenic, and celiac arteries, and the gastrohepatic and phrenoesophageal ligaments were divided with LigaSure™. Thus, the right crus of the diaphragm was exposed, the anterior and posterior branches of the vagus were divided, and the gastric tube was created with sequential firings of the 60-mm linear stapler along the lesser curvature of the stomach. Reconstruction following proximal gastrectomy was performed either intraabdominally or within the chest; in cases where a sufficient proximal margin could not be achieved during abdominal dissection, intrathoracic anastomosis was performed. In these cases, right thoracotomy was performed along the sixth intercostal space, the thoracic esophagus was dissected and transected below the azygos vein, and esophagogastric anastomosis was performed with a 25-mm circular stapler.

## Results

A total of 25 patients underwent laparoscopic D2 gastrectomy between May 2016 and May 2018. The patient characteristics are listed in Table 1. The study included 18 male patients (72%) and 7 female patients (28%) with a mean age of 49.8 ± 10.7. The majority of patients [18(72%)] underwent distal gastrectomy: four patients (16%) underwent total gastrectomy, while proximal gastrectomy was performed in three patients (12%).

**Table 1:** Patient characteristics (*n* = 25)

Patient characteristics	Values
Age (years) (mean ± SD)	49.8 ± 10.7
<50 [*n* (%)]	13 (52)
>50 [*n* (%)]	12 (48)
Sex	
Male [*n* (%)]	18 (72)
Female [*n* (%)]	7 (28)
Comorbidities	9 (36)
DM [*n* (%)]	4 (16)
HCV + ve [*n* (%)]	2 (8)
HTN [*n* (%)]	3 (12)
Tumor location	
Upper third (cardia, fundus) [*n* (%)]	4 (16)
Middle third (body) [*n* (%)]	3 (12)
Lower third (pylorus) [*n* (%)]	18 (72)
Neoadjuvant therapy [*n* (%)]	8 (32)
Type of gastrectomy	
Proximal gastrectomy [*n* (%)]	3 (12)
Total gastrectomy [*n* (%)]	4 (16)
Distal gastrectomy [*n* (%)]	18 (72)

*SD* standard deviation

The histopathological data are presented in Table 2. According to the AJCC classification (8th edition), 15 patients (60%) were in stages I and II, and 10 patients (40%) were in stage III. The median number of retrieved lymph nodes was 18 (range 5–35), and the median number of positive lymph nodes was 5 (range 1–21). A total of 13 patients had positive lymph nodes, while lymph nodes were negative in 12 patients. The proportion of patients with nodal involvement was 52%, and that of patients with no nodal involvement was 48%.

**Table 2:** Histopathological data (*n* = 25)

Variables	Values
Number of retrieved lymph nodes (median)	18 (5–35)
Adequacy of lymph node yield	
> 15 [*n* (%)]	15 (60)
< 15 [*n* (%)]	10 (40)
Positive proximal surgical margin [*n* (%)]	2 (8)
Positive distal surgical margin [*n* (%)]	0 (0)
Grade	
Grade 2 [*n* (%)]	18 (72)
Grade 3 [*n* (%)]	7 (28)
T stage	
p T1 [*n* (%)]	3 (12)
p T2 [*n* (%)]	8 (32)
p T3 [*n* (%)]	9 (36)
p T4a [*n* (%)]	5 (20)
N stage	
p N0 [*n* (%)]	12 (48)
p N1 [*n* (%)]	2 (8)
p N2 [*n* (%)]	5 (20)
p N3a [*n* (%)]	4 (16)
p N3b [*n* (%)]	2 (8)
TNM stage (AJCC 8th edition)	
IA [*n* (%)]	2 (8)
IB [*n* (%)]	5 (20)
IIA [*n* (%)]	4 (16)
IIB [*n* (%)]	4 (16)
IIIA [*n* (%)]	5 (20)
IIIB [*n* (%)]	4 (16)
IIIC [*n* (%)]	1 (4)
I + II stage [*n* (%)]	15 (60)
III stage [*n* (%)]	10 (40)
Postoperative chemoradiation [*n* (%)]	20 (80)

*AJCC* American Joint Committee on Cancer; *pN* pathological nodal stage; *pT* pathological T stage

Regarding the perioperative data (Table 3), the median operative time was 240 min (range 200–330), the median estimated blood loss was 250 ml (range 200–450), the median hospital stay was 8 days (range 6–30), and patients resumed oral fluids after a median period of 4 days (3–30). Conversion to open laparotomy was required in 7 patients (28%). The main cause of conversion was bleeding, which occurred in two patients. Other causes of conversion were injury to a visceral organ (transverse colon), adhesions, adherence of the mass to the head of the pancreas, proper assessment of the mass and technical difficulty (failure to fire the stapler). Reoperation was required in two patients: one as a result of anastomotic leakage and another because of duodenal stump leakage.

**Table 3:** Perioperative data (*n* = 25)

Variables	Values
Operative time (min) [*median* (*range*)]	240 (200-330)
Blood loss (ml) [*median* (*range*)]	250 (200-50)
Hospital stay (days) [*median* (*range*)]	8 (6-30)
Start oral fluids (days) [*median* (*range*)]	4 (3-30)
Conversion to open procedure [*n* (%)]	7 (28)
Bleeding [*n* (%)]	2 (8)
Adhesions between the stomach and colon [*n* (%)]	1 (4)
Proper assessment of the mass [*n* (%)]	1 (4)
Visceral organ injury [*n* (%)]	1 (4)
Mass was adherent to the pancreatic head [*n* (%)]	1 (4)
Failure to fire the stapler [*n* (%)]	1 (4)
Reconstruction	
Esophagogastrostomy [*n* (%)]	3 (12)
Billroth II [*n* (%)]	3 (12)
Roux-en-Y [*n* (%)]	19 (76)
Postoperative complications [*n* (%)]	4 (16)
Reoperation	2 (8)
Anastomotic leakage [*n* (%)]	1 (4)
Duodenal stump leakage [*n* (%)]	1 (4)

In terms of postoperative morbidity and mortality (Table 4), the overall morbidity and mortality rates among all patients were 16% and 8%, respectively. Three patients had surgical complications including anastomotic leakage, duodenal stump leakage, and intra-abdominal abscess. Nonsurgical complications were detected in one patient who developed postoperative acute myocardial infarction. Two mortalities were reported in the postoperative period: one patient died from severe sepsis because of duodenal stump leakage, and the other patient died because of acute myocardial infarction.

**Table 4:** Postoperative complications

Complications	*n* (%)
Morbidity	4 (16)
Anastomotic leakage (C-D grade IIIB)	1 (4)
Duodenal stump leakage (C-D grade V)	1 (4)
Intra-abdominal abscess (C-D grade IIIA)	1 (4)
Acute myocardial infarction (C-D grade V)	1 (4)
30-day mortality	2 (8)
Sepsis	1 (4)
Acute myocardial infarction	1 (4)

*C-D* Clavien-Dindo classification

## Discussion

Many studies have investigated the results of laparoscopic gastrectomy [3, 4, 14–16]. However, these studies could not provide an answer to whether laparoscopic D2 gastrectomy could be performed with the same quality as in open procedures. The current study reported the outcomes of laparoscopic D2 gastrectomy and confirmed that laparoscopic D2 gastrectomy can be safely performed regarding short-term oncological outcomes.

The operative time is longer in laparoscopic gastrectomy than in conventional open gastrectomy. Changing the instruments, cleaning the cameras, performing a mini-laparotomy and then creating the pneumoperitoneum during the anastomosis are responsible for increasing the operative time [5]. According to the literature, the operative time ranges between 196 min and 370 min for laparoscopic gastrectomy compared with a range from 168 min to 264 min for open gastrectomy [6, 7, 11, 12, 16, 17]. In our study, the median operative time was 240 min [range (200–330 min)], which is longer than that of the study conducted by Lee and colleagues in Korea, which reported a mean operative time of 227 min [11]. Additionally, this time is longer than that of the study conducted in China (CLASS-01 trial) [12], which demonstrated a mean operative time of 217 min. However, the operative time decreased with subsequent cases after more experience was gained. Moreover, the results of these studies were mainly reported for distal gastrectomies, which had a shorter operative time than total gastrectomies. On the other hand, the operative time in our study favorably compares with the results of studies performed in Japan (JCOG0912 trial) as well as the study conducted in Korea (COACT 1001 trial), which had mean operative times of 278 min and 257.4 min, respectively [3, 8].

Laparoscopic surgery has an advantage over open surgery in minimizing blood loss due to the rapid identification and control of small vessels [10]. In past reports, the estimated blood loss ranged from 82 ml to 333 ml for laparoscopic gastrectomy and 201–440 ml for open procedures [7, 16–19]. In the present study, the median estimated blood loss was 250 ml, which is considered similar to these studies. However, it is considered significantly higher than the blood loss in other studies conducted in Korea, China, and Japan. The mean blood loss was 153 ml in the KLASS-02 trial and was 105 ml and 115 ml in the CLASS-01 and JCOG0912 trials, respectively [3, 11, 12].

In terms of the adequacy of lymph node yield, in previous studies, the number of harvested lymph nodes ranged from 14–46.5 [3, 5, 8, 11, 12, 20–22]. The median number of retrieved lymph nodes in our study was 18, which was the same as that in the study conducted by Brenkman et al. [21] in the Netherlands. However, it was significantly lower than studies conducted in Asian countries. Lee and colleagues reported a mean number of 46.5 lymph nodes (KLASS-02 trial) [11]. In the CLASS-01 trial conducted in China [12], the mean number of retrieved lymph nodes was 36.1, while in the JCOG0912 trial performed in Japan, the median number of lymph nodes was 39 [3]. The superiority of these results from Asian studies in contrast to studies in western countries or to our study may be caused by a high qualification system in Asian countries. Additionally, in our study, 8 patients (32%) had received neoadjuvant chemotherapy, which may have influenced the number of harvested lymph nodes. A study in China concluded that neoadjuvant chemotherapy resulted in a reduced lymph node count [23]. This may be a potential reason for the decreased lymph node yield in the current study in comparison to the results from Korea and Japan. Nevertheless, dissection of 18 lymph nodes is considered adequate for tumor clearance and staging. In the current study, the overall rate of adequate lymph node staging (> 15 lymph nodes) was 60% and if we excluded the patients who received neoadjuvant chemotherapy, this rate increased to 70.6%. Furthermore, none of our patients developed nodal recurrence during follow-up. In the current study, the R0 resection in all procedures was 92%, which compared favorably to the study performed by Brenkman et al. [21] that reported an R0 resection rate of 90%. However, it is unfavorably comparable to the study conducted in Korea, which reported an R0 resection rate of 98.1% [11].

Laparoscopic gastrectomy is associated with lower morbidity rates than open gastrectomy. In the KLASS-02 trial, the overall complication rates of open and laparoscopic gastrectomy were 24.1% and 16.6%, respectively [11]. The difference was mainly caused by the decrease in local complications, not by systemic complications; in particular, the incidence of fluid collection and intra-abdominal bleeding was significantly lower with laparoscopic gastrectomy than with open surgery. The postoperative morbidity rates of laparoscopic gastrectomy range from 6.4 % to 24.2 % [5, 11, 12, 15, 16, 24]. In the current study, the morbidity rate was 16%, which is within the range reported previously, indicating that the morbidity rate in our study is satisfactory. Anastomotic leakage, which is considered a major complication of gastric surgery, should be evaluated appropriately. Anastomotic leakage rates from previous studies range from 0.2% to 14% [3, 5, 11, 12, 22, 24]. In our study, anastomotic leakage occurred in one case (4%) of total gastrectomy, which is within the range of other studies. Furthermore, D2 lymphadenectomy was performed in all cases in the current study, which is in contrast to most previous studies in which D2 gastrectomy was not routinely performed. Duodenal stump leakage is one of the most severe postgastrectomy complications with rates ranging from 0.4% to 2.4% in previous studies [5, 7, 25, 26]. In the present study, duodenal stump leakage was detected in one patient (4%) with a rate that was 1% higher than previously reported results. In this case, the patient developed sepsis, and reoperation was performed. Drains were inserted, but unfortunately, the patient died from complications. Intra-abdominal collection was found in one patient (4%) in the present study, and it was managed with radiological-guided drainage. This result was unfavorable to the study conducted by Katai et al. in Japan, which reported an intra-abdominal collection in 1.8% of cases [3]. On the other hand, the pancreatic fistula was not reported in the current study, and the incidence of the pancreatic fistula in other studies ranges from 0.4% to 1.9% [3, 11, 12]. Reoperation was indicated in two patients (8%), one of whom underwent distal gastrectomy and then developed duodenal stump leakage; the other patient underwent total gastrectomy and Roux-en-Y esophagojejunostomy and developed leakage from the anastomotic site. Abdominal exploration was performed, and the insertion of drains and a feeding jejunostomy was performed. Two weeks later, the fistula closed with conservative measures. The reoperation rate in our study was higher than that in studies conducted in Japan, Korea, and China, which demonstrated much lower reoperation rates. This is considered to be particularly due to the lack of sufficient experience in comparison to these highly qualified systems in these countries. On the other hand, the reoperation rate in our study was better than that of a study in Italy, which reported a reoperation rate of 9.52% [27].

According to the literature, mortality rates range from 0% to 6% [5, 11, 22, 24]. In our study, there were two mortalities (8%), which is slightly higher than in previous studies. The reason may be that one of these two mortalities was due to a nonsurgical cause; the patient developed acute myocardial infarction and did not respond to treatment. If we had excluded this patient, the actual surgical mortality would be only one patient (4%) and would be within the range of the previous studies. This patient underwent distal gastrectomy, developed duodenal stump leakage and died from severe sepsis. Although our results in terms of mortality are almost equivalent to other reported results, most of these other studies performed D1 resection rather than D2 resection, which had a higher incidence of morbidities and more technical complexity than D1 resection.

In addition to postoperative complications, conversion to an open procedure is another important index of quality. In the literature, rates of conversion are substantially different between the East and the West. Eastern studies reported a conversion rate ranging from 2.2% to 7% [3, 5, 11, 12, 18, 28]. On the other hand, Ecker et al. [20] and Brenkman et al. [24] reported conversion rates of 23.9% and 10%, respectively. In the present study, the conversion rate was 28%, which is significantly higher than that of other studies; however, the reason may be that most of these studies conducted D1 rather than D2 lymphadenectomy. In addition, this may be due to our early experience with this demanding technique; most of the conversions occurred in the first 10 patients; in subsequent patients, the rate of conversion decreased. The main cause for conversion was bleeding, which occurred in two patients (8%); the other causes were adhesions, mass-related causes (the mass was adherent to the pancreas), injury of a visceral organ or technical difficulty (failure to fire the stapler).

In terms of the postoperative period, in the present study, patients started oral fluids after a median period of 4 days, which is considered similar to the study performed in Korea, where patients started oral fluids after 3.7 days [11]. Additionally, this period is considered shorter than the results of two studies done in China that reported periods of 5.5 and 4.7 days prior to starting oral fluids [7, 12]. The early resumption of oral fluids without the need for imaging studies, mainly in cases of distal gastrectomy and a lower incidence of postoperative ileus, may be the reason for our shorter period to resume oral fluids in comparison to these studies.

Patients in our study had a median operative stay of 8 days, which is similar to the studies conducted in Japan, and Korea that reported a mean postoperative stay of 7.5 and 8.1 days, respectively [5, 11]. Patients in the current study had a shorter postoperative stay than those in the CLASS-01 trial, which reported a mean operative stay of 10.8 days [12].

Concerning the learning curve, proper patient selection resulted in improving the learning curve and decreasing the rates of conversion. Accordingly, the initial stages of laparoscopic gastrectomy should begin with the selection of patients with a body mass index (BMI) < 30 kg/m^2^ who are suitable for distal gastrectomy and should be conducted under the guidance of expert laparoscopic surgeons [29]. In the present study, conversion rates decreased from 4 conversions in the first 10 patients (40%) to 3 conversions in the last 15 patients (20%), and the median lymph node yield increased from 14 lymph nodes to 20 lymph nodes in the last 15 patients. Moreover, the median operative time decreased by 30 min in the last 15 patients.

There are some limitations in our study. First, we compared our results with the results of open and laparoscopic gastrectomy in the literature. Additionally, the study was limited by the small sample size. Finally, we did not evaluate the long-term outcomes, which might confirm the noninferiority of laparoscopic D2 lymphadenectomy in terms of survival analysis. Despite these limitations, the results are considered satisfactory in comparison to the results of other studies.

## Conclusions

Laparoscopic D2 gastrectomy is safe and feasible, and it can be performed by experienced surgeons to achieve optimal oncological short-term outcomes. However, more cases are needed with a sufficient follow-up period to analyze long-term outcomes.

## Data Availability

The datasets used and/or analyzed during the current study are available from the corresponding author on reasonable request.

## Abbreviations

AJCC: American Joint Committee on Cancer
BMI: Body mass index
C-D: Clavien-Dindo classification
CHA: Common hepatic artery
CV: Coronary vein
ECOG: Eastern Cooperative Oncology Group
EUS: Endoscopic ultrasound
LGA: Left gastric artery
LN: Lymph node
pN: Pathological nodal stage
pT: Pathological T stage
SA: Splenic artery
SD: Standard deviation
